# Inhibition of mitochondrial 2-oxoglutarate dehydrogenase impairs viability of cancer cells in a cell-specific metabolism-dependent manner

**DOI:** 10.18632/oncotarget.8387

**Published:** 2016-03-26

**Authors:** Victoria I. Bunik, Garik Mkrtchyan, Aneta Grabarska, Henry Oppermann, Danilo Daloso, Wagner L. Araujo, Malgorzata Juszczak, Wojciech Rzeski, Lucien Bettendorff, Alisdair R. Fernie, Jürgen Meixensberger, Andrzej Stepulak, Frank Gaunitz

**Affiliations:** ^1^ Belozersky Institute and Bioinformatics, Lomonosov Moscow State University, Moscow, Russia; ^2^ Faculty of Bioengineering and Bioinformatics, Lomonosov Moscow State University, Moscow, Russia; ^3^ Department of Biochemistry and Molecular Biology, Medical University of Lublin, Lublin, Poland; ^4^ Department of Neurosurgery, University Hospital Leipzig, Leipzig, Germany; ^5^ Max-Planck-Institute of Molecular Plant Physiology, Potsdam-Golm, Germany; ^6^ Max-Planck Partner Group at The Departamento de Biologia Vegetal, Universida de Federal de Viçosa, Viçosa, Brazil; ^7^ Department of Medical Biology, Institute of Agricultural Medicine, Lublin, Poland; ^8^ Department of Virology and Immunology, Institute of Microbiology and Biotechnology, Maria Curie-Skłodowska University, Lublin, Poland; ^9^ GIGA-Neurosciences, University of Liege, Liege, Belgium; ^10^ Department of Otolaryngology, MSW Hospital, Lublin, Poland

**Keywords:** amino acid transamination, cystine/glutamate antiporter, glioblastoma, 2-oxoglutarate dehydrogenase, succinyl phosphonate

## Abstract

2-Oxoglutarate dehydrogenase (OGDH) of the tricarboxylic acid (TCA) cycle is often implied to be inactive in cancer, but this was not experimentally tested. We addressed the question through specific inhibition of OGDH by succinyl phosphonate (SP). SP action on different cancer cells was investigated using indicators of cellular viability and reactive oxygen species (ROS), metabolic profiling and transcriptomics. Relative sensitivity of various cancer cells to SP changed with increasing SP exposure and could differ in the ATP- and NAD(P)H-based assays. Glioblastoma responses to SP revealed metabolic sub-types increasing or decreasing cellular ATP/NAD(P)H ratio under OGDH inhibition. Cancer cell homeostasis was perturbed also when viability indicators were SP-resistant, e.g. in U87 and N2A cells. The transcriptomics database analysis showed that the SP-sensitive cells, such as A549 and T98G, exhibit the lowest expression of OGDH compared to other TCA cycle enzymes, associated with higher expression of affiliated pathways utilizing 2-oxoglutarate. Metabolic profiling confirmed the dependence of cellular SP reactivity on cell-specific expression of the pathways. Thus, oxidative decarboxylation of 2-oxoglutarate is significant for the interdependent homeostasis of NAD(P)H, ATP, ROS and key metabolites in various cancer cells. Assessment of cell-specific responses to OGDH inhibition is of diagnostic value for anticancer strategies.

## INTRODUCTION

Increased thiamin transport in cancer cells emphasizes the role of thiamin in cancer cell metabolism [1, 2]. However, the significance of the function of the thiamin diphosphate (ThDP)-dependent enzymes of central metabolism in cancer has not been fully clarified. The isoform of ThDP-dependent transketolase, TKTL1, overexpressed in tumors, has long been believed to increase ribose-5-phosphate and NADPH required for intense proliferation [3-6], but turned out to be catalytically incompetent [7, 8]. Independent cellular studies failed to establish a correlation between the over-expression of TKTL1 or transketolase activity and proliferation of cancer cells [9, 10]. The ThDP-dependent pyruvate dehydrogenase (PDH) is often supposed to be down-regulated in cancer due to phosphorylation [3-5, 11, 12] under the control of the tumor regulators Myc and HIF-1 [13, 14]. In line with these findings, an inhibitor of the PDH kinase, dichloroacetate, has been shown to negatively affect tumor growth, which correlated with PDH activation [15-17]. However, our recent study revealed that active PDH is required for viability of certain glioblastoma cells, although the metabolic impact of PDH may significantly vary between different types of glioblastoma cells [18]. As a result, the increased thiamin uptake by tumors heightens questions regarding the significance for cancer cells of the third ThDP-dependent enzyme of central metabolism, 2-oxoglutarate dehydrogenase (OGDH). Although this multi-enzyme complex is known to be an essential regulatory point in the TCA cycle, controlling the glutamine and glutamate metabolism and limiting the metabolic flux through the cycle under a number of experimental settings [19-22], the OGDH-involving part of the TCA cycle from isocitrate to malate is often considered to be switched off in tumors. This is thought to occur due to the loss-of-function mutations of succinate dehydrogenase and fumarate hydratase [23, 24] downstream of OGDH and gain-of-function mutations of the non-TCA cycle isocitrate dehydrogenase isoforms 1 and 2, reducing the OGDH substrate 2-oxoglutarate to 2-hydroxyglutarate [11, 25, 26]. Interestingly, a catalytically inactive analog of lipoic acid, currently in clinical trials, induced cancer cell death concomitant with inhibition of the mitochondrial PDH and OGDH complex [27-29]. However, neither a specific contribution of PDH and OGDH, nor a potential involvement of other lipoate-reactive systems [30, 31], including the thioredoxin system [32, 33], which is a known target of anti-cancer therapies [34], can be resolved using the lipoic acid analog. The lack of specific knowledge on the role of OGDH in cancer metabolism and the recent development of the OGDH inhibitors selectively targeting the enzyme *in vivo* [20-22], prompted us to study the role of OGDH in cancer cell viability using the phosphonate analog of 2-oxoglutarate, succinyl phosphonate (SP). Binding to the enzyme as a tight transition-state analog [35, 36], SP inhibits OGDH, the first rate-limiting component of the mitochondrial multi-enzyme complex of oxidative decarboxylation of 2-oxoglutarate, in a highly selective and efficient manner. This was demonstrated using different approaches in a number of *in vivo* and cellular (*in situ*) systems [20, 22]. Given these properties, application of SP to cells aids our understanding of pathophysiological mechanisms upon perturbation of mitochondrial oxidation of 2-oxoglutarate. Taking into account the great variety and plasticity amongst tumor cells [37] and current evidence that cancer cells retain functional mitochondria and the capacity for oxidative phosphorylation in spite of the Warburg effect [12, 38], we screened the reactivity to SP of a number of cell lines with different levels of malignancy, including cells derived from the most common and malignant brain tumor, glioblastoma multiforme [39, 40]. An essential conclusion of this part of our study is that in many types of malignant cells including glioblastoma cells which frequently possess mutations of the TCA cycle enzymes up- and downstream of OGDH, the TCA cycle has a functional OGDH complex and is not interrupted at the isocitrate dehydrogenase step. In addition to different viability indicators, we used metabolic profiling and transcriptomics data deposited in the Gene Expression Omnibus (GEO) database. The complementary approaches enabled us to identify biochemical network, contributing to the cell-specific impact of OGDH inhibition on viability. The gained knowledge on molecular mechanisms of metabolic heterogeneity of cancer cells may be further employed to more efficiently fight tumorigenicity and drug resistance *in vivo*.

## RESULTS

### SP negatively affects viability of cancer cells as revealed by an NAD(P)H-dependent assay

**Figure 1:**
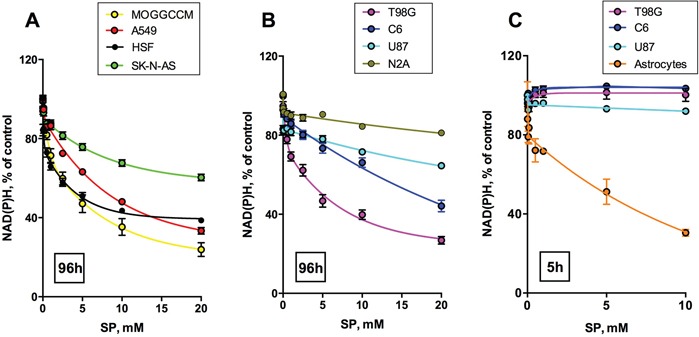
Concentration dependence of cellular viability on the OGDH inhibitor SP Legends on the graphs specify the cell lines and color code used. Values are means ± SEM from ≥ 3 independent experiments. Experimental curves were approximated by the biphasic exponential decay equations with parameters given in Table 1. **A, B.** Different cancer cell lines and primary human skin fibroblasts (HSF) were incubated for 96 h in the presence of different concentrations of SP in rich medium, and cellular NAD(P)H:MTT oxidoreductase activity was assayed as viability indicator. **C.** For a comparison of transformed (C6 rat glioma) and non-transformed (rat astrocytes) glial cells, short incubation times (5 h) in SP-supplemented HBSS medium were used.

Figure 1 shows a dose-dependent response of cancer cell lines to treatment with SP in the assay of cellular NAD(P)H-dependent reductase activity with a tetrazolium dye (MTT), which is used as an indicator of cellular viability. The activity is proportional to cellular reducing potential expressed as the level of cellular NAD(P)H, owing to which we will subsequently refer to the assay as estimating cellular NAD(P)H levels. For comparison of human cancer and non-cancer cells, also the SP action on primary human skin fibroblasts (HSF) is shown in Figure 1A. The data indicate that some of the human cancer cells, such as MOGGCCM, A549 and T98G, possess a reactivity towards SP which is similar to non-transformed human cells, where OGDH belongs to the central metabolism (HSF, Figure 1A). To compare SP reactivity of transformed and non-transformed cells from neural tissue, we included data from transformed cell lines from rodents (glioma C6 and neuroblastoma N2A), as normal human neurons and glial cells are not available. In particular, as seen from Figure 1C, NAD(P)H levels of rat astrocytes are significantly decreased by SP already after a short (5 h) exposure. During this time, glioblastoma cell lines of either human or rodent origin are not affected even in the minimal medium (Figure 3C). Worth noting, the decrease in the reducing potential of astrocytes was not accompanied by any comparable change in cellular protein. When the NAD(P)H level of astrocytes decreased more than 2-fold (Figure 1C, 5 mM SP), a 15% reduction in the astrocytic protein did not reach statistical significance. Thus, SP decreased the astrocytic NAD(P)H /NAD^+^ ratio rather than cell number. As a result, both cancer and non-transformed cells greatly vary in their reactivity to SP tested in the NAD(P)H-dependent viability assays.

**Figure 2:**
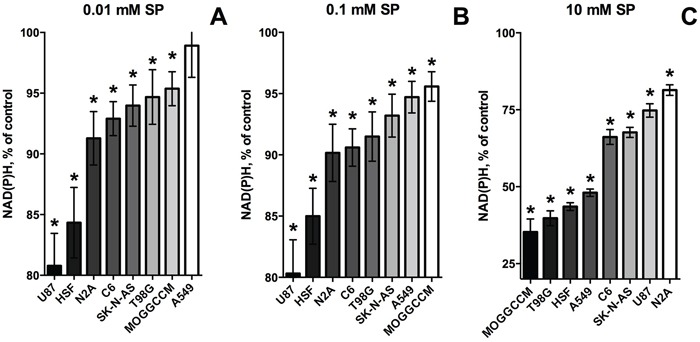
Change in relative sensitivity of different cell lines to SP with increasing SP concentrations Data of Figure 1A and 1B were used to range the cell lines according to the strength of their response to the SP exposure (96 h). The strength is colored in shades of grey. Statistical significance of the decreases in viability against the corresponding controls (*, p≤0.05) was determined by two-way analysis of variance (ANOVA) followed by Tukey test for multiple comparisons. **A.** SP 0.01 mM. **B.** SP 0.1 mM. **C.** SP 10 mM.

**Figure 3:**
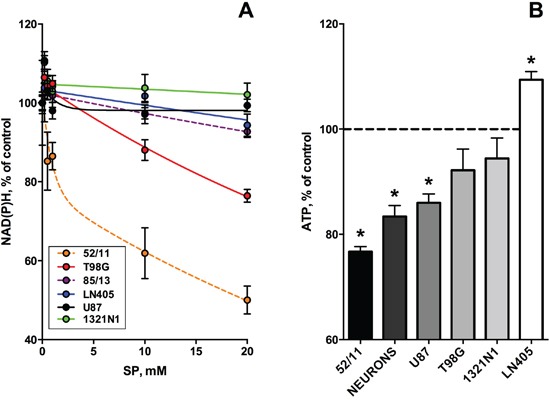
Dependence of viabilities of different glioblastoma cells on the OGDH inhibitor SP Cells were incubated for 24 h in the SP-supplemented rich medium. Experimental points are presented as means ± SEM. Four independent experiments with each cell line (1321N1, U87, LN405, T98G) were performed. As the primary human glioblastoma cells changed morphology after freezing-thawing, only the results obtained with the non-frozen cells are shown. **A.** CellTiterBlue assay of cellular NAD(P)H:resazurin oxidoreductase activity. In the color code-specifying legend the cell lines are presented in the order of decreasing cellular sensitivity to SP. Dashed lines show the results of single experiments with the freshly isolated primary glioblastoma cells (85/13 and 52/11). Experimental curves were approximated by the biphasic exponential decay equation with parameters given in Table 1. **B.** CellTiterGlo test of cellular ATP levels. Cellular sensitivity to SP is marked in shades of grey.

Non-linear regression analysis indicated that the viability decay kinetics is complex. In the cases of the well-expressed decay, the kinetic curves were best approximated by a biphasic process to a plateau value (Table 1, 96 h), which could become zero in case of high sensitivity to SP, exhibited by primary astrocytes (Table 1, 5 h). For the SP-resistant cells, which did not show significant viability decreases in 96 h, the plateau value was set to zero for the approximation procedure, but the biphasic kinetics remained valid for these cells as well (Table 1, 96 h, zero values marked by asterisks). The first phase of the SP-induced viability decays corresponds to 10-20% of the total viability, which is lost rapidly, followed by at least an order of magnitude slower decay. Such a kinetics suggests that the primary decrease in cellular NAD(P)H, caused by the OGDH inhibition, is addressed by the activation of alternative pathways of NAD(P)H production. This activation slows down the viability decay kinetics, resulting in the decreased rates of the decays in the second phase of the concentration dependences (Table 1, 96 h). Remarkably, increasing the SP concentration changed the relative sensitivities of different cells to SP (Figure 2). At SP≤0.1 mM (Figure 2A, 2B) U87 cells are even more reactive than HSF, while A549, MOGGCCM and T98G are resistant to SP action. However, at SP≥1 mM U87 cells become less susceptible than the A549, MOGGCCM and T98G cells, which, in contrast, acquire a high sensitivity to SP, comparable to that of HSF (Figure 2C, Figure 1). The change in the relative SP resistance with increasing SP concentration suggests varied abilities of different cells to adapt to the OGDH inhibition by SP.

**Table 1:** Parameters of the non-linear regression analysis of the viability (V) decay upon incubation of cells with SP

Time, assay	Cell line	Parameters of the viability decay regression					
		V_0_	V_fast_	V_slow_	k_fast_, mM^−1^	k_slow_, mM^−1^	Plateau
96 h, MTT	SK-NA-S	98	8	33	14	0.12	53
	A549	100	9	64	8	0.12	27
	C6	101	11	89	112	0.03	0*
	N2A	94	-	19	-	0.1	75*
	U87	100	19	82	~2*10^5^	0.01	0*
	HSF	96	21	36	9	0.24	39
	T98G	98	23	51	3	0.14	24
	MOGGCCM	99	26	53	1.6	0.13	20
5 h, MTT	Primary rat astrocytes	100	20	80	90	0.09	0
24 h, CTB	LN405	103	-	103	-	0.004	0*
	1321N1	105	-	105	-	0.001	0*
	U87	105	7	-	0.8	-	98*
	T98G	105	-	76	-	0.02	28*
	52/11	102	24	78	1.1	0.02	0*
	85/13	102	-	56	-	0.01	46*

Experimental curves in Figures 1 and 3 with significant viability decays were best approximated by the biphasic exponential equation: V_SP_=V_fast_*e^−k^_fast_^*[SP]^ + V_slow_*e^−k^_slow_^*[SP]^ + Plateau, where V_fast_=(V_0_-Plateau)*PercentFast*0.01; V_slow_=(V_0_-Plateau)*(100-PercentFast)*0.01. Plateau is V at infinite times; PercentFast is the fast fraction of the span from V_0_ to Plateau. For the SP-resistant cell lines exhibiting low amplitude of decay under experimental conditions, plateau could not be defined. In these cases (marked as *) the decay equations were simplified by manual setting the plateau value to zero. Then, if the regression constant k_slow_ approached zero, the corresponding V_slow_ was considered as plateau. The residual fraction (V_fast_) was considered as the fast or slow step according to the value of the regression constant k_fast_. The highly variable k_fast_ and differences between the regression parameter V_0_ and 100%, comparable to V_fast_, result from insufficient resolution of the fast process by regression analysis due to experimental limitations.

The next series of experiments focused on the most malignant type of glioma (WHO grade IV), glioblastoma, as these cells are often presumed to have no metabolic flux through OGDH [11, 23-26]. In addition to several cell lines derived from glioblastoma multiforme, we also used primary cancer cells isolated from human glioblastoma. In the latter case, we employed only freshly isolated cells at a low number of passages, because after freezing and thawing the cells changed morphology and growth properties. To better understand the initial metabolic damage induced by SP, we tried to limit the possibility of cellular adaptation by reducing the SP exposure time to 24 h. The choice of this time point took into account that the glioblastoma cell lines incubated with SP for 96 h, exhibited a distinct adaptive response, decreasing their SP reactivity with incubation time (Figure 1A, 1B; Table 1, 96 h). On the other hand, after incubation with SP for 5 h, the NAD(P)H levels of the glioblastoma cells did not change even in the minimal medium (Figure 1C). In view of some studies which showed that relative sensitivity of cells to treatments may depend on the viability test applied [41, 42], we also assessed the relative sensitivity of the glioblastoma cells to SP by different viability tests commercially available. As seen in Figure 3A, using the NAD(P)H-dependent CellTiterBlue viability test, we confirmed a lower resistance to SP of T98G cells, compared to U87 cells, as observed in the NAD(P)H-dependent MTT test (Figure 1B). The viability decrease in these different NAD(P)H-dependent tests exhibited the expected time dependence. That is, at 20 mM SP viability of T98G cells was reduced less in 24 h of the treatment (down to 80% of control, Figure 3A), compared to 96 h (down to 35% of control, Figure 1B). However, kinetic analysis of the viability decays at 96 and 24 h showed that decreases in the decay constants for the rapid and slow phases (k_fast_ and k_slow_, Table 1) were associated with the changes in the amplitudes of the rapid and slow phases (V_fast_ and V_slow_, Table 1). Thus, reduction of the time of SP exposure did not allow one to resolve the fast and slow phases. Their interdependence supports the assumption that the phases are due to the fast initial decrease in NAD(P)H by SP, which is slowed down by cellular adaptations at increasing SP exposures.

In Figure 3B, viability was assessed by determining cellular ATP levels, using the highly sensitive luminescent CellTiterGlo assay. This ATP-based assay was earlier shown to be a more sensitive indicator of the SP reactivity of primary neurons, compared to the NAD(P)H-dependent assay [42]. However, as seen from Figure 3B, also according to the CellTiterGlo assay, primary cerebellar granule neurons from rat do not significantly differ from the glioblastoma cells in their SP reactivity. As a result, the data presented in Figures 1-3 indicated that oncotransformation in different types of cancer including glioblastoma, does not necessarily exclude the high regulatory significance of the OGDH-catalyzed reaction, inherent in normal metabolism [19-22].

### Non-equal sensitivity of the glioblastoma NAD(P)H and ATP levels to SP delineates different metabolic types of glioblastoma

**Figure 4:**
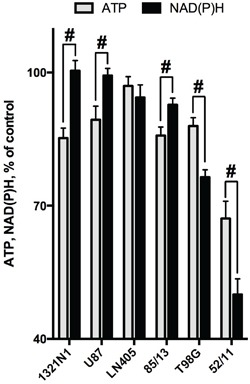
Comparison of the SP-induced changes in viabilities of glioblastoma cells, as determined by assays dependent on ATP (CellTiterGlo) or NAD(P)H (CellTtiterBlue) Cultured cells were incubated in rich medium supplemented with 20 mM SP for 24 h. The cells are ordered according to their ability to preserve the NAD(P)H levels. The data are presented as means ± SEM. Number of experiments as in Figure 3. Statistical significance of the differences between the two assays (#, p≤0.05) was determined by two-way ANOVA followed by Tukey test for multiple comparisons. The same statistical analysis of the SP-affected vs control cells revealed that levels of ATP in all cells but LN405 were different from those in the corresponding controls.

As shown above, the different viability tests based on assessing the NAD(P)H levels (MTT and CellTiterBlue) gave coinciding results. However, the responses of the glioblastoma ATP and NAD(P)H levels to SP treatment were not necessarily equal. For instance, the amplitude of the viability impairment of the primary glioblastoma cells (52/11) was lower when ATP levels were assayed. On the other hand, in U87 cells, a statistically significant 15% decrease in ATP levels was detected (Figure 3B), although NAD(P)H levels remained unperturbed under the same conditions (Figure 3A). Comparison of sensitivity to SP of cellular ATP and NAD(P)H in Figure 4 demonstrates that some glioblastoma cells exhibited a higher sensitivity in the ATP assay (U87, 1321N1, 85/13) and others in the NAD(P)H-based assay (T98G, 52/11). Assuming the ATP/NAD(P)H ratio as a cellular indicator of the oxidative phosphorylation efficiency, one could use the SP treatment to delineate the two metabolic sub-types of glioblastoma. These sub-types show a different interaction between TCA cycle impairment and oxidative phosphorylation. The glioblastoma cells increasing the ATP/NAD(P)H ratio under SP exposure (Figure 4, T98G and 52/11 cells) respond to the TCA cycle impairment by a higher transformation of the oxidation-derived energy (NAD(P)H) into the biosynthetic energy (ATP). In contrast, the glioblastoma cells decreasing the ATP/NAD(P)H ratio upon SP exposure (Figure 4, U87, 1321N1 and 85/13 cells) accumulate NAD(P)H rather than convert it to ATP, when the TCA cycle is impaired.

**Figure 5:**
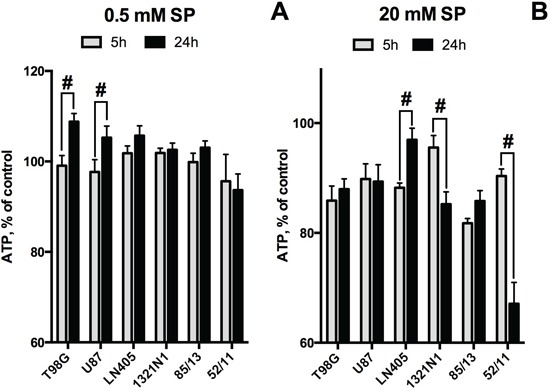
Time dependence of cellular ATP levels at low and high concentrations of SP **A.** SP 0.5 mM. **B.** SP 20 mM. At both SP concentrations, the cell order corresponds to the cellular ability to preserve ATP levels after 24 h at 0.5 mM SP. Values are means ± SEM from 4 independent experiments with cell lines. For primary glioblastoma cells 85/13 and 52/11 one independent experiment was done with freshly isolated cells, as cell morphology changed after freezing-thawing. Statistical significance (#, p<0.05) between the differences in ATP at 5 and 24 h is shown, as determined by two-way ANOVA followed by Tukey test. Compared to the corresponding controls, 20 mM SP induced statistically significant decreases in ATP levels in all cases except for 1321N1 and 52/11 at 5h, and LN405 at 24 h.

A study of the time-dependence of the SP-induced changes in cellular ATP levels demonstrated intermediary increases in cellular ATP during the exposure to SP (Figure 5), which has previously also been observed in primary neurons [42]. As seen in Figure 5A, increasing the incubation time from 5 to 24 h in the presence of 0.5 mM SP revealed ATP increases in T98G and U87 cells. However, at 20 mM SP, U87 and T98G cells could only preserve their ATP levels between 5 and 24 h, whereas a similar increase in ATP could be observed in LN405 cells which were more resistant to SP (Figure 5B). Remarkably, 1321N1 and 52/11 cells, which strongly differ in their sensitivity to SP according to the NAD(P)H-dependent assay (Figure 3A), were both unable to stabilize their ATP levels, as revealed upon incubation of 1321N1 and 52/11 cells with 20 mM SP (Figure 5B). This finding indicates that the high cellular NAD(P)H levels are not necessarily indicative of unperturbed metabolism.

The observed differences in the SP sensitivity of the glioblastoma NAD(P)H and ATP levels did not correlate with the glioblastoma cell growth rates. At the beginning of the SP exposure, the cells were usually at about 60% confluence. The determination of ATP levels in control cells, i.e. those incubated for 24 h without SP and providing for reference values in Figure 5, allowed us to estimate growth of the different cultures used in the experiment, between 5 and 24 hours. The corresponding increases in ATP levels were between 0% (85/13) and 60% (1321/N1). These proliferation indices did not reflect the SP sensitivity of the NAD(P)H levels of the cells. In particular, the SP sensitivity of NAD(P)H levels in both 85/13 and 1321/N1 cells was similarly low (Figure 3A, Figure 4). On the other hand, the SP-sensitive T98G cells and the SP-resistant LN405 cells (Figure 3A), which also significantly differed in the ability to preserve their ATP levels (Figure 5), showed a similar 30% growth under control conditions during the experiment. As a result, the time- and concentration-dependent changes in cellular ATP levels and ATP/NAD(P)H ratios due to SP treatment do not correlate with the cell growth rate, but depend on cell-specific molecular mechanisms employed to compensate for perturbation in the OGDH reaction.

### Correlation of the cancer cell sensitivity to SP with transcriptomics data

**Figure 6:**
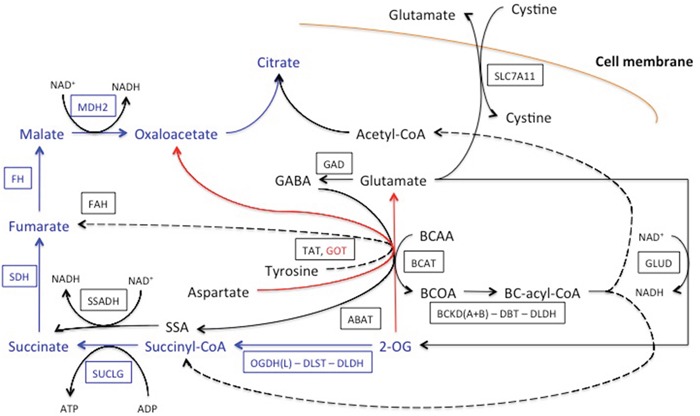
Network of the OGDH(L)-related reactions, shunting the TCA cycle block by the OGDH(L) inhibitor SP The relevant part of the TCA cycle is shown in blue, the major transamination reaction by aspartate aminotransferase (GOT) is shown in red. Other pathways linked to OGDH(L) and affiliated with the TCA cycle are in black. They include transamination of 2-oxoglutarate (2-OG) with branched chain 2-amino acids (BCAA) to branched chain 2-oxo acids (BCOA), with gamma-aminobutyric acid (GABA) to succinyl semialdehyde (SSA) and with tyrosine. The transamination products are further oxidized to the branched chain acyl-CoA (BC-acyl-CoA), succinyl-CoA, acetyl-CoA, succinate and fumarate, as shown in the scheme. Glutamate accumulated due to the transmination of 2-oxoglutarate, may be oxidized to 2-oxoglutarate or exchanged for extracellular cystine. The enzymes and cystine/glutamate antiporter are given by human gene names, as in Table 2, and framed. Dotted lines denote multiple reactions in the pathways. See text for other details.

Cell-specific significance of the OGDH reaction for the viability parameters may be assessed by cellular expression of the enzymatic components of the OGDH multi-enzyme complex and enzymes of the OGDH-linked network (Figure 6), which are indicators of the functional activity of different OGDH-dependent pathways. Our analysis of expression of particular enzymes of interest used the global transcriptomic data deposited in the Gene Expression Omnibus (GEO) database (see Methods). The combined expression of the two functionally competent isoforms of 2-oxoglutarate dehydrogenase (further denoted as OGDH(L)), coded by the OGDH (house-keeping isoform) and OGDHL (brain isoform) genes [43, 44], was considered for estimation of the OGDH function. In the following section, we will mainly refer to the relative transcript abundances given in Table 2 by the enzyme/gene names only. Relative abundance of the functional 2-oxoglutarate dehydrogenase multi-enzyme complex in the cell lines was estimated as the ratio of transcripts of genes coding for the rate-limiting component OGDH(L) (OGDH and OGDHL genes) and the second substrate-specific component dihydrolipoyl succinyltransferase (DLST gene). The four cell lines for which the results of several different experiments on gene expression were found in GEO (A549, T98G, SK-NA-S, U87), were compared, taking into account the higher sensitivity to SP of A549 and T98G vs SK-NA-S and U87 (Figure 1A and 1B). The relative sensitivities in the NAD(P)H-based assay (Figure 1) were chosen for the comparison, assuming the NAD(P)H levels to be more direct indicators of the inhibition of the NADH-producing OGDH than the ATP levels. The colored pattern of Table 2 exposes not only the similarity in the abundance of the OGDH(L)-related gene transcripts in the SP-sensitive lines A549 and T98G, but also the difference between these SP-sensitive lines and those rather resistant to SP, i.e. SK-NA-S and U87. First of all, abundances of the OGDH(L)-DLST sub-complex were often shifted to lower values in A549 cells (from 0.05 to 0.7) and T98G cells (abundance coefficients from 0.3 to 1.3), compared to SK-NA-S cells in culture (coinciding values 1.1) and U87 (abundance coefficients from 0.8 to 3). The lower abundances of the OGDH(L)-DLST sub-complex in A549 and T98G correlate with a higher susceptibility of NAD(P)H levels in these cell lines to SP (Figures 1-3). As shown earlier, cellular viability depends on the spare OGDH threshold capacity [45]. Obviously, the threshold is easier reached by the OGDH(L) inhibition in cells with lower levels of the functional OGDH(L) multi-enzyme complex, explaining the observed correlation between the OGDH(L) expression and the SP sensitivity. The cell-specific impact of the OGDH(L) function may also be seen from the relative expression of OGDH(L) compared to other enzymes of the TCA cycle. In fact, in the cell lines A549 and T98G which are more sensitive to SP, the lower abundance of the functional OGDH(L)-DLST sub-complex (Table 2, yellow pattern) coincides with the lowest abundance of OGDH(L) compared to other TCA cycle enzymes (Table 2, blue pattern). In contrast, the cells more resistant to SP (SK-N-AS and U87), were not characterized by the minimal transcript abundance of OGDH(L) among the TCA cycle enzymes. In these SP-resistant cells, the TCA cycle “bottle-neck” is shifted upstream of OGDH(L), to isocitrate dehydrogenase or, according to some experiments with U87 cells, to citrate synthase (Table 2, blue pattern). Remarkably, according to the TCA cycle expression analysis, relative OGDH(L) abundance in SK-N-AS cells differs in the cell culture and tumors grown *in vivo* (SK-N-AS xenografts) (Table 2). The difference suggests a condition-dependent shift of the TCA cycle “bottle-neck” to OGDH(L) in xenografts, i.e. *in vivo*.

**Table 2:** Assessment of the relative expression of OGDH(L) and its network proteins in the cell lines differing in their resistance to SP (SK-N-AS and U87 are more resistant than A549 and T98G, Figure 1)

Enzymes and pathways	Ratio of transcripts	SK-N-AS xenografts	SK-N-AS		A549			T98G		U87	
			n=2	n=4	n=3	n=1	n=1	n=3	n=1	n=3	n=2
OGDHC	OGDH(L)/DLST	0.5	1.1	1.1	0.7	0.3	0.05	0.3	1.3	0.8	3
TCA cycle	OGDH(L)/OGDH(L)	1	1	1	1	1	1	1	1	1	1
	SUCLG1.2/OGDH(L)	3.9	1.9	8	15	8	78	16	22	9	0.7
	SDH(A+B)/OGDH(L)	2.3	1.9	7	10	4	39	10	7	6	3
	FH/OGDH(L)	3.7	1.1	8	9	4	70	13	15	4	0.5
	MDH2/OGDH(L)	9.0	1.3	21	27	9	127	7	16	10	3
	CS/OGDH(L)	5.6	1	9	11	5	44	22	12	5	0.05
	IDH2/OGDH(L)	2.7	0.8	0.6	4	1.4	6	4	2	0.6	0.3
Transaminases using OG	GOT(1+2)/OGDH(L)	4	2	6	12	6	38	16	9	6	1
	BCAT(1+2)/OGDH(L)	1	1	2	5	2	10	2	14	0.8	0.3
	ABAT/OGDH(L)	0.1	0.3	0.3	2	0.1	0.4	0.4	0.1	0.1	0.004
	TAT/OGDH(L)	0.1	0.3	0.05	0.2	0.02	0.1	0.1	1	0.4	0.01
BCKADH	BCKD A+B/OGDH(L)	1	1	1	2	1	5	1	1	1	0.5
	DBT/OGDH(L)	0.3	1	3	4	3	1	13	10	1	0.01
	DLDH/OGDH(L)	3	1	13	12	4	62	11	10	6	0.6
Glu metabolism	GLUD(1+2)/OGDH(L)	1	2	2	4	4	37	7	5	4	1
	GLUL/OGDH(L)	1	1	6	1	0.1	2	11	6	2	0.1
	GLS/OGDH(L)	2	1	4	4	2	23	4	7	2	0.1
	GLS2/OGDH(L)	0.01	0.2	0.04	0.01	0.01	0.2	0.2	0.3	0.04	0.004
	SLC7A11/OGDH(L)	0.1	1	1	11	6	72	9	15	1	0.005
GABA shunt	GAD(1+2)/OGDH(L)	0.1	0.5	0.1	0.1	0.1	0.2	0.4	0.4	0.6	0.2
	SSADH/OGDH(L)	0.3	1	1	1	1	6	0.2	1.0	0.01	0.004
Tyr degredation	FAH/OGDH(L)	0.2	1	2	2	1	6	4	0.4	3	1
	AACS/OGDH(L)	1	1	2	2	1	7	1	4	1	0.2
Glu receptors (ionotropic)	GRIA/OGDH(L)	0.4	1	0.1	0.1	0.3	0.2	0.6	0.1	0.1	0.05
	GRIN/OGDH(L)	0.3	2	1	1	0.3	1	1	2	1	3

The GEO database was used to extract the transcript values as described in Methods. Enzymes and cystine/glutamate antiporter SLC7A11 are given by the abbreviations used for human gene names. The corresponding reactions are shown in Figure 6, except for glutaminase (isoforms GLS, GLS2) and glutamine synthase (GLUL), which are omitted for simplicity. Signals of the gene transcripts from *n* datasets employed to obtain the data presented in the corresponding columns were averaged dependent on coincidence, as described in methods. Blue pattern shows relative abundance of the TCA cycle enzymes in each cell line (vertical comparison), with bright blue marking the transcript ratios in excess to the minimal one shown in pale blue. Yellow pattern refers to the comparison of SK-N-AS, A549, U87 and T98G (horizontal comparison), regarding the functional OGDH(L) subcomplex (OGDH(L)/DLST) and its network proteins. Intense yellow marks the range of higher expression ratios compared to the range of expression ratios in pale yellow. Some differences could be associated with the SP resistance only for T98G and U87 cells. For comparison between cells in culture and *in vivo*, the available experiment on SK-N-AS xenografts, obtained by inoculating SK-N-AS cells into nude mice (experiment under the GEO accession number GSM302678), is included.

Thus, according to the expression analysis, OGDH(L) may limit the TCA cycle flux in A549 and T98G cells, which increases their sensitivity to the OGDH(L) inhibition. In contrast, no limitation of the TCA cycle flux in the SK-N-AS and U87 cells corresponds to the lower dependence of these cells on the OGDH(L) inhibition by SP (Figure 1). As a result, the relative abundance of functional OGDH(L) correlates with cellular sensitivity to OGDH(L) inhibition.

Along with the different impact on cell viability of the OGDH reaction itself, the viability impairment due to perturbation in the OGDH(L)-catalyzed reaction will depend on cellular ability to shunt the inhibited reaction and/or activate alternative pathways of NAD(P)H or ATP production. The cell-specific networks (Figure 6) respond to the OGDH inhibition according to the mass law action. That is, accumulation of 2-oxoglutarate due to the OGDH(L) inhibition increases saturation of the enzymes and transporters of this network in accordance with their expression and substrate affinity, stimulating the transformations of 2-oxoglutarate beyond that inhibited by SP. It was previously shown in a variety of cells and tissues that the inhibition of OGDH(L) by SP increases the transamination of 2-oxoglutarate to glutamate and other amino acids. As a result, the degradation of amino acids, particularly those restoring the TCA cycle downstream of OGDH(L), is stimulated [42, 46, 47]. The closest to OGDH(L) restoration of the TCA cycle occurs through the dehydrogenase of the branched chain 2-oxo acids and GABA shunt, producing along with NADH also the downstream intermediates succinyl-CoA and succinate (Figure 6). Oxidative deamination of glutamate by glutamate dehydrogenase with the concomitant production of NADH, 2-oxoglutarate and NH_4_^+^ may restore 2-oxoglutarate, further increasing its transamination with the amino acids (Figure 6). Regarding potentially toxic NH_4_^+^ produced in this reaction, and the importance of glutaminase in cancer transformation [11], cellular abundances of glutamine synthase (GLUL) and glutaminase (isoforms GLS and GLS2) were also taken into consideration (Table 2), although for the sake of simplicity these reactions are not shown in Figure 6. The data presented in Table 2 indicates that many of the enzymes involved in these 2-oxoglutarate-linked reactions, are more abundant in the SP-sensitive (A549 and T98G) than in the SP-resistant (SK-N-SA and U87) cells. That is, metabolic networks with minimal OGDH(L) abundance potentially limiting the TCA cycle flux at the 2-oxoglutarate degradation step (Table 2, abundances in blue), are characterized by a higher expression of the pathways utilizing 2-oxoglutarate in other reactions (Table 2, abundances in yellow). When OGDH(L) is inhibited in such metabolic networks, these pathways may compensate for the inhibition by generating the products (NADH and succinyl-CoA) or the downstream intermediate (succinate) of the OGDH(L) overall reaction. However, the high expression of these compensatory pathways in A549 and T98G cells compared to SK-N-AS and U87 ones (Table 2), does not support cellular ability to preserve the NAD(P)H levels in the former compared to the latter, when cells are exposed to SP (Figure 1A and 1B).

Apart from dysfunctional metabolism of dicarboxylates, tyrosine degradation, initiated by the tyrosine transamination with 2-oxoglutarate [48-50], and glutamate exchange for cystine through x_c_^−^ cystine/glutamate antiporter SLC7A11 [51-54], are considered important for cancer cell metabolism. Stimulation of tyrosine degradation to fumarate and acetoacetate (terminal step catalyzed by FAH, Figure 6), with ATP-dependent acetoacetate activation to acetoacetyl-CoA by AACS (not shown in Figure 6), may occur due to SP-induced increase in tyrosine transamination with accumulated 2-oxoglutarate, catalyzed by TAT (Figure 6). However, abundances of the enzymes of the tyrosine degradation pathway (Table 2), where TAT is supposed to be rate-limiting, do not exhibit a clear correlation of this pathway with cellular resistance to SP. In contrast, along with other enzymes of glutamate metabolism, the cystine/glutamate antiporter SLC7A11 is additionally more abundant in cells with NAD(P)H levels less resistant to SP (A549 and T98G, Table 2).

Given that SP increases glutamate production from accumulated 2-oxoglutarate (Figures 8, 9), while glioblastoma cells may be impaired by glutamate excitotoxicity through ionotropic receptors [52], we also compared expression of these receptors as potential contributors to cellular sensitivity to SP. Indeed, Table 2 shows that the range of expression of the AMPA glutamate receptors (all isoforms of GRIA) was somewhat higher in the more sensitive T98G cells (abundances from 0.1 to 0.6), compared to resistant U87 cells (abundances from 0.05 to 0.1).

**Figure 7:**
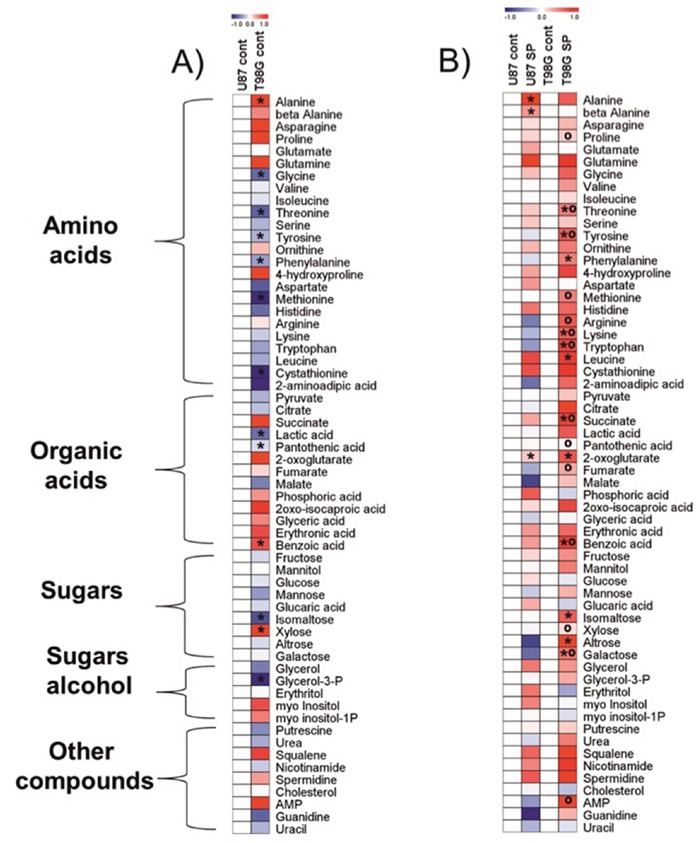
Heat map visualization of the relative metabolite levels in T98G and U87 cells Metabolites were determined as described in Methods.The color code of the heat maps is given at the log(2) scale above the diagrams. Extracts were obtained in at least five independent experiments. Statistical significance was determined by Student's t test (P < 0.05). Asterisks (*) indicate significant difference from the control shown as the blank map. Circles (°) indicate significant difference between the SP-treated T98G cells and U87 cells. **A.** Control cells. Metabolite levels in T98G cells were normalized to those of U87 cells. **B.** Cells treated with 0.5 mM SP for 24 h in rich medium. Metabolite levels after the SP treatments of T98G or U87 cells were normalized to the respective controls.

**Figure 8:**
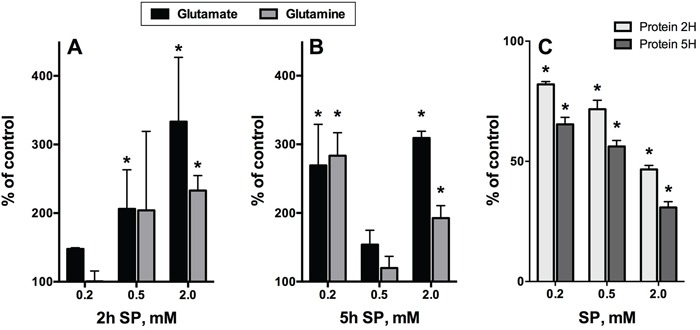
Time-dependent changes in the intracellular content of glutamate, glutamine and protein upon incubation of rat cerebellar granule neurons in the SP-supplemented growth medium The data were obtained using five independent neuronal cultures. Statistical significance was estimated using two-way analysis of variance (ANOVA) followed by Dunnett's test for multiple comparisons. Difference to the control values (p≤0.05) is indicated by asterisks. **A, B.** Glutamate and glutamine. **C.** Cellular protein.

**Figure 9:**
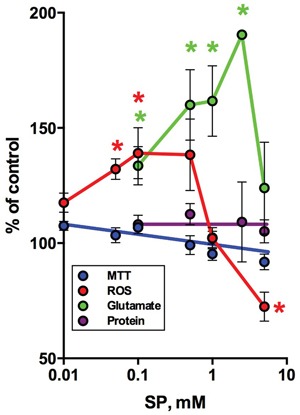
Changes in cellular NAD(P)H:MTT reductase activity, ROS, glutamate and protein after 5 h incubation of neuroblastoma N2A cells in SP-supplemented minimal medium Means ± SEM from three independent experiments are shown. Statistical significance was estimated using one-way analysis of variance (ANOVA) followed by Dunnett's test for multiple comparisons. Difference to the control values (p≤0.05) is indicated by asterisks.

### SP-induced changes in metabolic profiles of glioblastoma cells with different SP resistance in NAD(P)H-based viability tests

To understand molecular mechanisms of cellular responses to SP of glioblastoma cells, we also characterized cellular heterogeneity using metabolic profiling of glioblastoma cell lines with different SP resistance, i.e. U87 and T98G lines. Remarkably, metabolic profiles of these cells under control conditions were different. It is obvious from Figure 7A that, compared to U87, T98G cells have lower levels of most amino acids, with statistically significant decreases in glycine, threonine, tyrosine, phenylalanine, methionine and cystathionine. However, alanine was increased concomitant with decreased lactate, in T98G vs U87 cells. Metabolic difference between T98G and U87 cells is further supported by different levels of pantothenic and benzoic acid, isomaltose, xylose and glycerol-3-phosphate (Figure 7A).

Given the large differences in the metabolic profiles of T98G and U87 cell lines under control conditions, the metabolic changes observed in response to the SP treatment are expected to be cell-specific too, which was indeed observed (Figure 7B). In order to detect the primary metabolic changes induced by SP, we used the treatment conditions which did not significantly change the viability indicators, i.e. cells were incubated with 0.5 mM SP for 24 h. Already under this low SP concentration, metabolic perturbations were detectable in both the SP-sensitive T98G and SP-resistant U87 cells (Figure 7B). The signature of the SP inhibition of OGDH, an increase in 2-oxoglutarate [47], was well expressed in the two cell lines. In accordance with the SP effect on NAD(P)H levels (Figure 1, Figure 3A), the more sensitive T98G cell line exhibited many more changes after the treatment with SP, while in U87 cells only alanine and β-alanine increased significantly along with 2-oxoglutarate (Figure 7B). It must be noted, however, that even the limited changes in the U87 metabolic profile were indicative of serious failures, such as perturbed function of the TCA cycle (alanine, 2-oxoglutarate) and increased degradation of pyrimidine nucleotides (β-alanine). The different patterns of the SP-induced changes in the amino acid pool of the T98G and U87 cells reciprocated initial differences in the pool of the cells under control conditions. That is, the originally lower levels of many amino acids in T98G cells increased and became higher than in U87 cells. An important difference between the T98G and U87 cells was observed with regard to SP-induced changes in the TCA cycle intermediates beyond 2-oxoglutarate. Succinate and fumarate accumulated in T98G cells treated with SP much more than in U87 cells (Figure 7B). SP also significantly changed sugars, such as isomaltose, xylose, altrose and galactose in T98G cells compared to U87 cells. Furthermore, increased adenosine-5-monophosphate (AMP) was observed in T98G vs U87 cells after SP treatment. Thus, the original metabolic profiles of T98G and U87 cells (Figure 7A) and their responses to SP (Figure 7B) supported the cell-specific metabolism and different compensatory pathways suggested by the transcriptomics data (Table 2).

### Glutamine and glutamate as metabolic indicators of SP action in glial and neuronal cells

Because of efficient transamination between 2-oxoglutarate and glutamate, both compounds were indicators of the OGDH inhibition by SP in different systems [42, 47]. However, metabolic profiling of the SP-treated glioblastoma cells showed a much higher accumulation of glutamine, not glutamate (Figure 7). This was in good accordance with the known high activity of glutamine synthase (GLUL, Table 2) and the glutamate/cystine antiporter (Table 2), competing for the intracellular glutamate in glioblastoma cells [54]. In contrast, under similar conditions, SP-treated neurons were characterized by primary accumulation of glutamate, with the glutamine increase following, but never exceeding that of glutamate (Figure 8). The difference was the most obvious at low (0.2 mM SP for 2 h, Figure 8A) and high (2 mM SP for 5 h, Figure 8B) exposures of neurons to SP, confirming recent data on the presence of glutamine synthase not only in astrocytes, where the major part of the brain enzyme is localized, but also in neurons [55]. It was therefore of interest to determine if glutamate would increase in SP-exposed neuroblastoma cell lines. As seen in Figure 9, an up to 2-fold increase in glutamate was observed in neuroblastoma in the SP concentration-dependent manner. Moreover, in both neurons (Figure 8B) and neuroblastoma (Figure 9), the SP-induced changes in the level of glutamate exhibited a complex time dependence, with an initial increase in intracellular glutamate content followed by a decrease. The decreases in glutamate were not attributable to cell loss, because the levels of glutamate were normalized per protein content. It is worth to note however, that neuronal, but not neuroblastomal, protein was significantly reduced by SP (Figure 8C vs Figure 9). The perturbed neuronal metabolism associated with significant (down to 50%, Figure 8C) loss of cellular protein was probably responsible for a metabolic switch, leading to the secondary increase in intracellular glutamate at extensive SP exposure (for 5 h at 0.5 to 2 mM SP, Figure 8B).

### Increases in glutamate and reactive oxygen species (ROS) upon incubation of neuroblastoma N2A cells with SP

Some studies demonstrated that in neuroblastoma cells glutamate caused oxidative stress and mitochondria-mediated apoptosis [56, 57], reminiscent of the glutamate-induced excitotoxicity in neurons. Such neurotoxic action of glutamate in neuroblastoma was observed despite the significant decrease in expression of the NMDA receptors in brain cancer cell lines [58]. We therefore checked if glutamate accumulation in neuroblastoma exposed to SP was associated with changed ROS levels. As seen from Figure 9, a significant increase in glutamate upon incubation of N2A cells with SP was paralleled by an increase in superoxide anion radical production (reactive oxygen species, ROS), as detected by lucigenin chemiluminescence, already at a concentration of 0.1 mM SP. However, a further increase in SP concentration (from 0.1 to 2.5 mM) decreased ROS, although the glutamate increased up to 2-fold before decreasing at high SP (5 mM) (Figure 8, green line). Crucially, all the changes occurred before any significant decay in cellular viability or cell loss could be detected by the MTT test or protein assay (Figure 9). The statistically significant decrease in viability, corresponding to a 15% decrease in the NAD(P)H-dependent MTT-reductase activity, was observed only following 5 h incubation with 10 mM SP. Under these conditions, ROS showed up to a 2-fold decrease from control values (not shown), suggesting that cellular NAD(P)H may be consumed by ROS-scavenging reactions. Thus, our study pointed to a biphasic kinetic of the SP-induced changes in the glutamate and ROS levels in neuroblastoma, with a decrease in accumulated ROS followed by a decrease in cellular reducing power.

## DISCUSSION

### Oxidative decarboxylation of 2-oxoglutarate is significant for tumor cell metabolism and survival

Cancer cells are widely known to increase their glycolysis under normoxic conditions, which led Otto Warburg to hypothesize that mitochondria and oxidative phosphorylation are impaired in cancer. Although this hypothesis was not supported by the following studies which showed that in the majority of cancer cells mitochondria are functional and the capacity for oxidative phosphorylation normal [12, 38], the flux through the mitochondrial TCA cycle in cancer remains under debate, as the cycle is often supposed to be truncated at the isocitrate dehydrogenase step upstream of OGDH. Using selective inhibition of OGDH(L) by SP allowed us to reveal the metabolic significance of the OGDH(L)-catalyzed reaction in a variety of cancer cells. This finding has specific importance in view of the controversial suggestions of the glutaminolysis-coupled pathways, discussed in the literature. Although glutaminolysis in normal cells is known to require OGDH, cancer cells frequently have mutations in the TCA cycle enzymes succinate dehydrogenase and fumarate hydratase [59] downstream of OGDH, and also reduce the OGDH substrate 2-oxoglutarate to 2-hydroxyglutarate [26]. Owing to this, glutaminolysis in cancer cells was suggested to occur through the isocitrate dehydrogenase-dependent carboxylation of 2-oxoglutarate [25, 60, 61]. However, this interpretation of isotope tracer studies was not supported by independent studies [54, 62, 63] and is thermodynamically impossible because of a low intracellular concentration of gaseous CO_2_. Indeed, mammalian carboxylation needs ATP and biotin for the CO_2_ activation, and carboxylation of 2-oxoglutarate by the recombinant tumor mutants of isocitrate dehydrogenase has never been observed, even if the mutants do reduce 2-oxoglutarate to 2-hydroxyglutarate [26]. Analogous mutants of the isocitrate dehydrogenase homolog oxidizing homoisocitrate did not catalyze the carboxylation reaction either [64]. Our findings provide experimental evidence for the functional importance of the OGDH-dependent turnover of the TCA cycle in cancer cells, clearly showing that homeostasis of different types of tumor cells, including the most malignant glioblastoma cells, is significantly perturbed upon inhibition of oxidative decarboxylation of 2-oxoglutarate. However, the dramatic changes in cellular homeostasis due to SP treatment (Figures 7, 9) were poorly expressed in the viability tests, based on either ATP or NAD(P)H assays, at short exposure times (Figures 3, 9, [42]). Obviously, detection of differences in cellular proliferation by such assays implies a long incubation time, whereas metabolic indicators are best suited to detect homeostatic perturbations before they are translated into cell number.

We also show here that the cell-specific differences in sensitivity of the viability parameters to the OGDH(L) inhibition correlate with potential limitation of the TCA cycle flux by OGDH(L) (Table 2). The correlation between the gene transcript abundances (Table 2), sensitivity of cellular NAD(P)H to the OGDH(L) inhibition (Figures 1–3) and the SP-induced changes in metabolic profiles (Figure 7) supports the notion that the transcript abundances and protein expression are optimal for biologically relevant protein-protein interactions [65].

Both the OGDH(L) multi-enzyme complex and the TCA cycle metabolon are known as essential regulatory systems of cellular metabolism. The OGDH(L) complex has often been shown to limit the TCA cycle flux, especially under maximal metabolic load, whereas the 2-oxoglutarate/glutamate ratio is key for the retrograde signaling from mitochondria to nucleus [19-22]. The physiological significance of the TCA cycle flux limitation by OGDH(L) is supported by the observed shift of the TCA cycle enzyme with minimal transcript abundance from isocitrate dehydrogenase to OGDH(L), when the independent database-deposited experiments on SK-N-AS cells in culture are compared to the cells within tumors growing in mice (SK-N-AS xenografts) (Table 2). The shift in minimal transcript abundance may change the TCA cycle rate-limiting step, causing the known differences in the TCA cycle flux of cancer cells *in vivo* and *in situ*, particularly in utilization of glutamine [63]. As a result, tumor cells *in vivo* could be more sensitive to the OGDH(L) inhibition, compared to the same cells in culture. This is supported by the high sensitivity of the primary glioblastoma cells 52/11 to the OGDHC inhibition (Figure 3).

### Comparison of SP action in normal and tumor cells

In this work, we have shown that both the normal and malignant cells may exhibit different reactivity to the OGDHC inhibition, with the reactivity also dependent on the assays employed. However, oncotransformation *per se* is not associated with insensitivity to the OGDH inhibition. Besides, the cell-specific metabolism results in cell-specific markers of SP reactivity. For instance, in neuronal cells SP causes a 2-fold (neuroblastoma, Figure 9) or a 3-fold (cerebellar granule neurons, Figure 8) increases in glutamate, while in glioblastoma cells the changes in glutamate are not expressed, whereas glutamine increases about 2-fold (Figure 7). Moreover, similar changes in the same markers may be associated with different consequences for cellular homeostasis. That is, the similar SP-induced increases in glutamate of cultured primary neurons (Figure 8) and neuroblastoma cells (Figure 9) are observed together with a drastic difference at the protein level, which is strongly reduced by SP in neurons (Figure 8C), but not in neuroblastoma cells (Figure 9). A comparison with the published data also shows that, when SP acted on hippocampal neurons, their ROS production first decreased (at 0.2 mM SP), followed by an increase (at 0.5 mM SP) [66]. As shown in Figure 9, neuroblastoma N2A cells exhibited an opposite concentration dependence on SP: initial ROS increase at SP < 0.2 mM is followed by a decrease at SP > 0.2 mM. Also in cervical cancer cells, down-regulation of the OGDH(L) gene was associated with a decrease in ROS [67], which we observe upon strong inhibition of OGDH(L) at SP > 0.2 mM in neuroblastoma (Figure 9). Further confirming the biological significance of the interplay between the OGDH(L) function and cellular ROS production [66-70], these findings exhibit essential differences between neurons and neuroblastoma cells regarding the interplay, obviously dependent on the cell-specific metabolic networks of compensatory reactions (Figure 6, Table 2). The network may also contribute to the cell-specific manifestations of the glutamate-induced excitotoxicity. In some experimental settings, the tumor cells used glutamate to increase their proliferation [53, 58, 71]. Other studies revealed glutamate to induce oxidative stress and mitochondria-mediated apoptosis in neuroblastoma cells [56, 57], although these processes were not dependent on NMDA receptors mediating the glutamate excitotoxicity in neurons. In glioblastoma, glutamate could induce necrosis through ionotropic glutamate receptors and impairment of the cystine/glutamate antiporter [52]. Our data on increased expression of GRIA receptors and the cystine/glutamate antiporter in T98G vs U87 glioblastoma cells (Table 2) agrees with a stronger sensitivity of the reducing power of T98G to SP (Figures 1–3). Thus, multiple mechanisms of glutamate action inside and outside cells, cell-specific protein expression and variations in experimental settings may all contribute to the glutamate-induced outcome for cellular viability, followed by the dose-response curves *in situ*. Therefore, only understanding of molecular mechanisms involved in toxic drug effects helps translating the results of cellular research to *in vivo* conditions.

### Specific inhibition of key enzymes as a tool to decipher molecular mechanisms of cancer cell heterogeneity: significance for therapeutic strategies

Transcriptomics (Table 2) and metabolic profiling under control conditions (Figure 7A) and when responding to drugs (Figure 7B) provide complementary data to decipher molecular mechanisms of heterogeneity of cancer cells. The differences between metabolic profiles of T98G and U87 cells (Figure 7A) are in good accordance with the transcriptomics data (Table 2), additionally supported by a 5-fold lower activity of the OGDH complex in T98G compared to U87 cells [72]. First, correlated differences in the TCA cycle intermediates, i.e. higher levels of 2-oxoglutarate and succinate, accompanied by lower levels of malate, pyruvate and citrate in T98G vs U87 (Figure 7A), agree with OGDH limiting the TCA cycle flux in T98G, but not in U87 cells. Second, a lower content of many amino acids in T98G vs U87 cells (Figure 7A) may be due to increased steady-state transamination and oxidation of amino acids in T98G (Table 2). Third, a higher expression of the pathways metabolizing amino acids and generating succinate in T98G cells (Table 2) is confirmed by a significantly higher elevation of the amino acid pool and accumulation of succinate, observed in the SP-treated T98G compared to U87 cells (Figure 7B). Remarkably, neither the restoration of the TCA cycle, nor the NADH generation in the compensatory reactions (i.e. glutamate dehydrogenase, succinate semialdehyde dehydrogenase and branched chain 2-oxo acid dehydrogenase, Figure 6) occurring in T98G cells exposed to SP, are able to support the control level of cellular reducing power to the same extent as in SP-treated U87 cells, where these pathways are less expressed. This non-trivial finding indicates that the metabolic impact of the same intermediates (such as NADH, succinyl-CoA and succinate) arising through different pathways, is not equivalent. Other studies on glioblastoma cell lines arrived at similar conclusion regarding cellular metabolism of NAD(P)H [72] or glutamine [54]. Indeed, perturbed fluxes often result in metabolic disbalance causing increased ROS levels. Increased ROS generation upon the inhibition of OGDH(L) was observed in this (Figure 8, SP<0.2 mM) and other studies (reviewed in [22, 70]). Much stronger accumulation of succinate in T98G vs U87 cells exposed to SP (Figure 7B) suggests succinate dehydrogenase to be the SP-stimulated ROS source. Changed saturation of the enzyme with succinate and NADH, particularly when the malate-aspartate shuttle is perturbed and fumarate accumulated, was shown to increase the succinate dehydrogenase-dependent ROS production *in vivo* [73]. The conditions are apparently stimulated by SP due to excessive 2-oxoglutarate transamination with aspartate and tyrosine, on one hand, and production of succinate and NADH from GABA shunt and branched chain amino acids, on the other hand (Figure 6). All these compensatory pathways are less expressed in U87 vs T98G cells (Table 2), which may better preserve the NAD(P)H levels of U87, compared to T98G cells (Figures 1, 3). In addition, as pointed out above, the higher abundance of the cystine/glutamate antiporter SLC7A11 in cells of higher sensitivity to SP (T98G and A549 vs U87 and SK-N-AS, Table 2) may add to the NAD(P)H consumption by excessive cystine infux through the antiporter (Figure 6). When stimulated by glutamate accumulation in cells with inhibited OGDH(L), the intracellular cystine will consume NADPH for the reduction to cysteine, but further glutathione synthesis may be perturbed due to metabolic alterations resulting in decreased ATP levels. As a consequence, in spite of the ability of the 2-oxoglutarate-utilizing pathways beyond OGDH(L) to restore the TCA cycle by generating NADH, succinyl-CoA and succinate, perturbed fluxes would be anticipated to increase ROS. More NAD(P)H consumption for their scavenging would decrease the reducing potential of cells, reflected in the MTT and CellTiterBlue assays (Figures 1, 3). Disparity in cellular changes in ATP and NAD(P)H upon SP action (Figure 4) is well explained by the recently shown inhibition of ATP synthase through direct binding of 2-oxoglutarate to the enzyme β-subunit [74]. Accumulation of 2-oxoglutarate in both T98G and U87 cells may explain their similar sensitivity to SP in the ATP assay, although the cells react to SP differently in the NAD(P)H-based assay due to the reasons discussed above. Because the ATP synthase inhibition by 2-oxoglutarate is reversible, the observed cell-specific restoration of ATP levels during increased SP exposure (Figure 5) may be due to compensatory changes in the metabolic network addressing the 2-oxoglutarate accumulation.

Analysis of the existing and developing anticancer therapies has indicated that drugs have to be developed and tested for specific tumors, since drug effects in different types of cancer cells are not necessarily the same [15, 37]. As demonstrated in our study, synthetic compounds with a well-defined molecular mechanism of action are of great help in finding solutions for the problem. Indeed, different expression of the enzymes of the OGDH-dependent metabolic node (Figure 6, Table 2) superposed on varied resistance of different cancer cells to the OGDH inhibitor SP (Figures 1–5, 7) allows one to identify the cell-specific pathways to overcome the drug action. Based on this knowledge, combinations of drugs could be defined to increase cell specificity of metabolic impairment, including that in cancer vs healthy cells.

## MATERIALS AND METHODS

### Cell culture

Human Caucasian lung adenocarcinoma (A549), human neuroblastoma (SK-NA-S), human astrocytoma (MOG-G-CCM), and rat glioma (C6) cell lines were obtained from the European Collection of Cell Cultures (Center for Applied Microbiology and Research, Salisbury, UK). Mouse neuroblastoma N2A and human glioblastoma cell lines LN405, T98G, U87, 1321N1 were obtained from the European Collection of Cell Cultures or from the American Type Culture collection (LGC Standards GmbH; Wesel, Germany). The primary cell cultures 52/11 and 85/13 were obtained from human glioblastoma tissue according to established methods [75]. Human skin fibroblasts (HSF) were isolated by the outgrowth technique from skin explants [76]. Primary rat astrocytes and cerebellar granule neurons were obtained from rat pups using established protocols [42, 47]. The cultures were kept at 37°C in a humidified atmosphere of 95% air and 5% CO_2_. All cell culture media were supplemented with 10% FBS, penicillin (100 units/ml) and streptomycin (100 μg/ml) (Sigma, St. Louis, MO). Established cell culture-specific protocols were used for the culture maintenance. MOG-G-CCM and C6 cells were cultured in DMEM with 1 g/L glucose, 1 mM pyruvate and 4 mM L-glutamine. SK-NA-S cells and HSF were grown in a Nutrient mixture F-12 Ham with 3.2 g/L glucose, 0.5 mM pyruvate, 2.5 mM L-glutamine. A549 cells were grown in a 1:1 mixture of DMEM (D6046, Sigma) and Nutrient mixture F-12 Ham (D8437, Sigma). Glioblastoma cells were grown in DMEM with 4.5 g/L glucose and 2 mM glutamax. Astrocytes were grown in EMEM with 1.3 g/L glucose. Neuroblastoma N2A cells were grown in DMEM with 4.5 g/L glucose and 4 mM L-glutamine. Cerebellar granule neurons were grown in NBM medium with B27 supplement and 2 mM glutamax.

For the viability and ROS tests, cells were placed on transparent 96-well microplates (NUNC, Roskilde, Denmark) and white 96-well microplates (Corning Incorporated, New York, USA), correspondingly. Primary astrocytes and neurons were treated with SP after their maturation was complete, i.e. 10-14 days in culture for astrocytes [47] and 10-12 days in culture for cerebellar granule neurons [42]. Other cells were exposed to SP 24 h (96-well plates) or 48 h after plating (6-well plates). Density of these cells upon seeding was chosen so that cells were about 60-70% confluent at the beginning of the experiment, not reaching confluence by the end of incubation with SP.

### Cell viability/growth assay

MTT (Sigma Chemicals, St. Louis, MO, USA), CellTiterBlue and CellTiterGlo reagents (Promega, Mannheim, Germany) were employed to test cellular viability. If not indicated otherwise, serial dilutions of SP (0.01-20 mM) were added to cells in fresh culture media. During the medium exchange before the SP addition, glioblastoma cells were brought to DMEM with 1 g/L glucose, 1 mM pyruvate and 2 mM glutamax. The influence of SP in minimal medium was studied in the earlier employed buffered salt solution, pH 7.4, containing 135 mM NaCl, 5 mM KCl, 5 mM glucose, 1.8 mM CaCl_2_, 0.01 mM glycine, 20 mM 4-(2-hydroxyethyl)-1-piperazineethanesulfonic acid (HEPES) [42].

Viability of the cell lines was assessed after 96 h of exposure to SP by using the MTT method in which the yellow tetrazolium salt 3-(4,5-dimethylthiazole-2-yl)-2,5-diphenyltetrazolium bromide (MTT) is reduced by viable cells at the expense of their NAD(P)H to purple formazan crystals. Tumor cells were incubated for 3 h with MTT solution (5 mg/ml). Formazan crystals were solubilized overnight in SDS buffer (10% SDS in 0.01 N HCl) and the product was quantified spectrophotometrically by measuring absorbance at 570 nm using an Infinite M200 Pro microplate reader (Tecan, Männedorf, Switzerland). When viability of N2A cells was assessed after 5 h of exposure to SP, a modification of the MTT method described above was applied, adapted to the shorter time of the experiment. In this case, cells were incubated for 3 h with MTT solution (0.15 mg/ml), followed by the solubilization of formazan crystals during 5 min in isopropanol/HCl solution (20 ml of isopropanol and 0.12 ml of 12 N HCl). The product was quantified spectrophotometrically by measuring absorbance at 580 nm using a GLOMAX Multi+ detection system of Promega (Madison, WI, USA). In good accordance with the published application of both MTT procedures, we did not notice any significant change in the estimation of the N2A viability by the two MTT procedures applied. Viability of glioblastoma cells was also assessed using CellTiterBlue and CellTiterGlo reagents (Promega) according to the manufacturer's instructions with modifications described earlier [42]. To exclude a possible influence of the experimental medium components on the ATP assay system, the incubation media were exchanged for the SP-free ones. That is, the cell lysis in ATP assay was performed by substitution of the incubation media for 50 μL of the CellTiterGlo reagent diluted 1:1 with HBSS. Luminescence was detected after 10 min incubation. CellTiterBlue assay was earlier shown not to be affected by SP [42], owing to which 20 μL of the CellTiterBlue reagent were added directly into 100 μL of the media per well. Fluorescence was detected after 1 h incubation with the CellTiterBlue reagent.

### Measurement of ROS production

ROS were assayed by chemiluminescence of lucigenin after its reaction with superoxide anion radical according to [77]. After 5 h incubation of N2A cells with SP, the incubation medium was exchanged for 100 μL of cold phosphate-buffered saline (PBS) with 2 μM CaCl_2_ and 0.08 μM lucigenin, and chemiluminescence was measured, using the GLOMAX Multi+ detection system of Promega (Madison, WI, USA). The measurements were done right after addition of lucigenin, as repeated assays after 10 min incubation in the presence of lucigenin did not increase the luminescence. As a blank, chemiluminescence in control wells was used, where no cells were present, but the cell growth medium was exchanged for PBS with added calcium and lucigenin.

### Glutamate and glutamine assays

Neuroblastoma glutamate was assayed enzymatically using a commercially available preparation of bovine liver glutamate dehydrogenase (Sigma–Aldrich, Taufkirchen, Germany) as described previously [47]. Glutamate dehydrogenase (0.05 mg/mL, 45 units/mg) was added to 0.1 M Tris/HCl buffer, pH 8.0, containing 1.5 mM NAD^+^ and 0.02 mL of cell lysate. Quantification of glutamate was done from the maximal increase in optical density at 340 nm, using the molar extinction coefficient of NADH 6220 M^−1^ cm^−1^, based on equimolar production of NADH from glutamate in the glutamate dehydrogenase reaction. The cells on 6-well plates were exposed to 0.01-2.5 mM SP for 5 h at 37°C in the buffered salt solution [42], pH 7.4, containing 135 mM NaCl, 5 mM KCl, 5 mM glucose, 1.8 mM CaCl_2_, 0.01 mM glycine, 20 mM 4-(2-hydroxyethyl)-1-piperazineethanesulfonic acid (HEPES). After the incubation was complete, the plates were put on ice, medium was removed, the cells were washed twice with cold PBS and extracted with cold water solution containing 40% methanol supplemented with 0.12% acetic acid. The plates were slowly shaken for one hour. Cells were scraped, collected in 0.75 ml tubes and centrifuged at 6200 g for 15 min at 4°C. The pellets were stored at −20°C until protein analysis.

Neuronal glutamate and glutamine content was assayed by the GC-MS procedure described below under *Metabolic profiling*. The neuron-specific modifications for the extract preparation were as employed before [42].

### Metabolic profiling

1×10^6^ cells were grown on Petri dishes for 24 hours. Then the growth medium was changed to DMEM with 1g/L glucose containing 10% FBS, antibiotics, 2 mM glutamax with or without 0.5 mM SP. After an incubation time of 24 hours cells were washed once with ice-cold PBS, and subsequently the methanol extraction and metabolic profiling were performed according to [42]. Metabolites of both cell lines were extracted in 2 ml of ice-cold methanol containing 0.05 mM ribitol as internal standard for the relative quantification of metabolite abundance [78]. After centrifugation, the supernatant was collected and stored at -80°C until the analysis. The pellet was used for protein quantification according to [79]. Polar and non-polar phases of the extracts were separated by addition of water and chloroform. Aliquots of 0.3 mL of the polar phase (upper phase) were dried using a vacuum centrifugation concentrator (Eppendorf, Hamburg, Germany). Sample derivatization was carried out according to [78]. 40 μl of methoxyamine hydrochloride (CAS 593-56-6, Sigma, Munich, Germany) dissolved at 20 mg/mL in pure pyridine (CAS 110-86-1, Merck, Darmstadt, Germany) was added to the dried samples and maintained at 37°C for 2h. After a rapid centrifugation, 70 μl of *N*-methyl-*N*-(trimethylsilyl)-trifluoroacetamide (MSTFA, CAS 24589-78-4, Macherey & Nagel, Düren, Germany) were added and the samples were vortex mixed and maintained at 37°C for additional 30 min. The tubes were centrifuged and 70 μl were transferred to glass vials in order to be run in GC-MS. The samples were analyzed by GC-MS using two technical replicates per sample and where necessary were injected at two different dilutions in order to accurately measure both high and relatively low abundant metabolites. The chromatograms and mass spectra were evaluated by Chroma TOF 1.6 (Leco, St Joseph, MI) and TagFinder 4.0 [80] and metabolites were identified by comparison with mass spectral database [81, 82]. The GC-MS metabolite determinations were normalized to ribitol and protein content (μg) and presented as fold-changes to the respective controls. The metabolic profiles of U87 and T98G cells under control conditions were compared by normalization of the T98G metabolite levels to those in U87 cells. For the heat map visualisation the normalized values were Log(2) transformed and the heat map was created using multiple array view (MeV)^®^ software (http://www.tm4.org/mev.html). Five independent extracts for each cell line (control and SP-treated) were analysed. Statistical significance of the differences was analysed by Student's *t*-test at *p* < 0.05.

### Protein assay

Total cellular protein was determined by dissolving the cell pellet in 20 μL of 1 M KOH at 37°C for 60 min, followed by analysis with a BSA Protein Assay Kit (Thermo Scientific, Prod # 23232).

### Analysis of transcriptomics data

Transcriptomics data for the full annotated genes of interest were extracted from the NCBI Gene Expression Omnibus (GEO) database using Microarray Retriever [83]. The program output presents the data as an excel file of sorted genes with detailed information on each gene. For the four cell lines analyzed (A549, SK-N-AS, T98G and U87) global transcriptomics data were available. The following datasets were used in our analysis: for SK-N-AS - GSM302678, GSM302679, GSM302680, GSM302681, GSM1086183, GSM1086184, GSM558078, GSM558079, GSM558080, GSM692855; for A549 - GSM1414981, GSM1414982, GSM1414983, GSM94306, GSM1122064; for T98G - GSM96274, GSM96275, GSM96276, GSM211868; for U87 - GSM862922, GSM862923, GSM862924, GSM1280363, GSM1280365. Transcript values were grouped for averaging according to their coincidence, which depended on the microarray and evaluation algorithm used. The coincidence was high (StDev < 20%) in different experiments which used the same Affymetrix platform and validation algorithm. Using other microarrays (Agilent or different platforms of Affymetrix) and/or evaluation algorithms could result in transcript signals which differ far beyond StDev < 20%. Our comparison of cell lines was therefore based on the range of relative transcript values obtained from *n* coinciding experiments. The values were normalized to the level of expression of 2-oxoglutarate dehydrogenase in the same experiment(s). The expression of 2-oxoglutarate dehydrogenase was calculated as the sum of the signals corresponding to expression of the two catalytically competent isoforms of the enzyme, coded by the OGDH and OGDHL genes [43, 44].
